# Stress Memory and the Inevitable Effects of Drought: A Physiological Perspective

**DOI:** 10.3389/fpls.2016.00143

**Published:** 2016-02-15

**Authors:** Eva Fleta-Soriano, Sergi Munné-Bosch

**Affiliations:** Department of Plant Biology, Faculty of Biology, University of BarcelonaBarcelona, Spain

**Keywords:** drought stress, drought tolerance, long-term memory, photosynthesis and the environment, chloroplasts

## Abstract

Plants grow and develop by adjusting their physiology to changes in their environment. Changes in the abiotic environment occur over years, seasons, and days, but also over minutes and even seconds. In this ever-changing environment, plants may adjust their structure and function rapidly to optimize growth and reproduction. Plant responses to reiterated drought (i.e., repeated cycles of drought) differ from those to single incidences of drought; in fact, in nature, plants are usually exposed to repeated cycles of drought that differ in duration and intensity. Nowadays, there is increased interest in better understanding mechanisms of plant response to reiterated drought due, at least in part, to the discovery of epigenomic changes that trigger drought stress memory in plants. Beyond epigenomic changes, there are, however, other aspects that should be considered in the study of plant responses to reiterated drought: from changes in other “omics” approaches (transcriptomics, proteomics, and metabolomics), to changes in plant structure; all of which may help us to better understand plant stress memory and its underlying mechanisms. Here, we present an example in which reiterated drought affects the pigment composition of leaves in the ornamental plant *Silene dioica* and discuss the importance of structural changes (in this case in the photosynthetic apparatus) for the plant response to reiterated drought; they represent a stress imprint that can affect plant response to subsequent stress episodes. Emphasis is placed on the importance of considering structural changes, in addition to physiological adjustments at the “omics” level, to understand stress memory in plants better.

## Introduction

The environment is constantly changing, not only over seasons and years, as we currently experience with global warming effects, but also daily and even over a few minutes and sometimes seconds, as occurs with the variations in light intensity at dawn or dusk. Therefore, plants may adjust their metabolism, structure, and function rapidly to optimize growth and reproductive capacity at any given moment. At the same time, the capacity of plants to adjust the mechanisms at work in them to an ever-changing environment determines their capability to respond to future environmental conditions. Diurnal and seasonal cycles in climate conditions force plants to adjust their metabolism; and stress memory allows them to select, at least to some extent, the most appropriate response to certain changes in the environment. Thus, the capacity of plants to adjust the mechanisms that function within them to an ever-changing environment shapes their future fitness and ultimately makes it possible for plants to live in the great diversity of habitats they have colonized. Plant stress responses can be characterized by an initial alarm phase, in which mechanisms for coping with the stress are activated and growth-related processes slow down. This is generally followed by a resistance phase, in which the plant modulates its structure and function in ways that allow it to withstand the stress and repair any damage already caused. If the stress persists or if it is too severe, the plant dies; if the stress subsides, however, the plant may recover and can reach a new optimal physiological status in the recovery phase. Whatever the future holds for a plant, the first stress episode will leave an imprint on it that will affect its response to subsequent stresses.

It is possible to categorize plant responses to drought stress in accordance with the organizational level of study: from a more molecular perspective, where one can find the “omics” approach, to a more “classical” approach that includes structural changes. However, structural changes also occur at different levels of organization, from the whole plant (e.g., changes in the number of leaves, leaf area, or leaf thickness) to the genetic level (e.g., histone modification); so the two approaches overlap. Within the “omics” approach, we find changes in epigenomics, which affect DNA activity without modifying the gene sequence; transcriptomics, which are changes in gene expression; proteomics, referring to changes in proteins; and finally metabolomics, which are changes in metabolites (Singh et al., 2015). At the structural level, changes in the root/shoot biomass ratio, number of leaves, leaf area, leaf mass per area ratio (LMA), leaf size, and/or structure of the photosynthetic apparatus coupled to chloroplast organization and shape (Pallardy and Kozlowski, 2008; Aroca, 2012) may all also affect plant response to subsequent stresses.

Here, we discuss the importance of both “omics” and structural changes, and present an example in which reiterated drought affects the pigment composition of leaves in the ornamental plant *Silene dioica*. Structural changes that result from the plant response to reiterated drought may be considered important stress imprints that can affect plant response to subsequent stresses and should therefore be carefully considered, in addition to “omics” approaches, in the study of plant responses to reiterated drought or other abiotic stress factors.

## Drought Stress Memory In Plants

Of all the environmental stresses, drought is one that has the most negative effects on plant growth and development, and can lead to important losses of productivity capacity (Ciais et al., 2005). The effects of drought stress vary depending of many factors, such as the intensity and duration of the stress, the plant genotype or growth phase, and also the imprint previous stress episodes have left on the plant. This imprint, or stress memory, can be defined as the structural, genetic, and biochemical modifications that have occurred as a consequence of stress exposure and which make the plant more resistant (although it might also be more sensitive in some cases) to future exposure to the same stress factor (if the later stress is different, the term “cross-stress tolerance” is more appropriate). Although the increase in resistance may compromise plant productivity in the short term, for example through a reduction of photosynthesis, it represents increased tolerance to subsequent stress and therefore favors productivity in the long term (Bruce et al., 2007). If the stress is too severe, however, productivity may be negatively affected in both the short and long term.

Despite the fact that the mechanisms underlying the stress imprint or memory are still not fully understood, it has been shown that an accumulation of signaling compounds and transcription factors together with epigenomic modifications may play a major role in them (Bruce et al., 2007; Conrath et al., 2009). For example, it has been reported that abscisic acid (ABA) may be involved in drought stress memory in the short term, such as over days or weeks (Ding et al., 2012; Fleta-Soriano et al., 2015) and also that epigenomic changes play a role in aspects related to meristem functioning (Kaya et al., 2001) and seed development (Wu et al., 2000), which will in turn affect plant development and productivity in the long term.

Among the plethora of responses that plants have evolved to withstand drought stress, photoinhibition of photosynthesis occurs in several plant species and directly affects productivity in the short term. A reduction in the function of the photosynthetic electron transport chain causes an excess of energy in chloroplasts that may, among other consequences, lead to increased production of reactive oxygen species (ROS; Ghosh and Xu, 2014). Photoinhibition of photosynthesis in drought-stressed plants is preceded by an increase in ABA levels that leads to stomatal closure, which prevents dehydration. At the same time, however, ABA promotes the production of protective substances (e.g., osmolytes) and helps maintain membrane structure (Verslues et al., 2006), thereby regulating genes with ABA-response elements (ABREs) in their promoter region (Evers et al., 2010). This raises the following important questions. If the plant recovers from the water deficit, could such responses persist over time and benefit the plant if it is challenged again by a new period of drought? Will double-stressed plants respond differently from single-stressed plants? What methodological approaches can we use to understand the mechanisms underlying drought stress memory?

## “Omics”: New Challenges

Nowadays, there is increased interest in better understanding the mechanisms involved in plant responses to reiterated drought, in part due to the discovery of epigenomic changes. This discovery, in parallel with the ongoing development of massive gene expression analysis, such as that conducted using microarrays and deep sequencing, has revolutionized the field. Furthermore, proteomics and metabolomics have helped to solve the puzzle by providing important new insights into our understanding of plant responses to drought stress (Ruan and Silva, 2011).

Global warming has led to forecasts of an increase in drought in some areas of the world and, even more importantly, an increase in the areas potentially exposed to severe drought over the next few decades, due to an increase in temperatures of between 1.4 and 5.8°C by the end of the 21st century (Salinger, 2005). Global warming effects are, however, occurring at the same time as important advances in “omics” technology (epigenomics, transcriptomics, proteomics, and metabolomics). This technology provides us with very useful information that allows us to understand drought stress responses and the mechanisms underlying plant stress memory better. This in turn may lead to improved plant productivity under changing climatic conditions and could balance the possible losses due to the effects of global warming.

Abscisic acid is known to be involved in plant responses to reiterated drought. In some plant species, ABA levels are higher under drought conditions if the plants have previously been challenged by water deficit, that is in double-stressed plants than in single-stressed plants; thus indicating drought stress memory (Fleta-Soriano et al., 2015). Since ABA plays an essential role in plant responses to drought stress, important transcriptional effects can be assumed, since several genes contain ABREs in their promoter regions (Evers et al., 2010). Virlouvet and Fromm (2015) showed that previously stressed plants have stomatal apertures that remain partially closed during a recovery period, which reduces transpiration during subsequent dehydration stress. Interestingly, this response was associated with increased expression of *9-CIS-EPOXYCAROTENOID DIOXYGENASE 3* (*NCED3*) and *ALDEHYDE OXIDASE 3* (*AAO3*), which are key modulators of ABA biosynthesis. This is in agreement with a drought memory effect, in which ABA plays a key regulatory role.

Histone modifications and DNA methylation can trigger important changes in gene transcription (Chinnusamy and Zhu, 2009). Alterations in the chromatin structure, such as modifications of the histone H3K4me3 in rice, have been associated with changes in the expression of some genes related to drought stress (Chen et al., 2013). Meanwhile, it has been reported that DNA hypermethylation occurs in salt-stressed *Mesembryanthemum crystallinum* when metabolism shifts from C3 to CAM (Dyachenko et al., 2006) and also in the root tips of pea plants under drought stress (Labra et al., 2002). Therefore, changes in chromatin structure and DNA methylation are currently considered a general response not only to drought stress, but also to other abiotic stresses, conferring both stress memory and cross-stress tolerance (reviewed by Urano et al., 2010; Kim et al., 2015). However, whether or not changes in chromatin structure and DNA methylation are modulated by ABA in drought stress memory is still to be determined. Furthermore, more research is needed to determine the effects these epigenomic changes trigger at the proteomic and metabolomic levels.

## Structural Changes: A More “Classical” Perspective?

**FIGURE 1:**
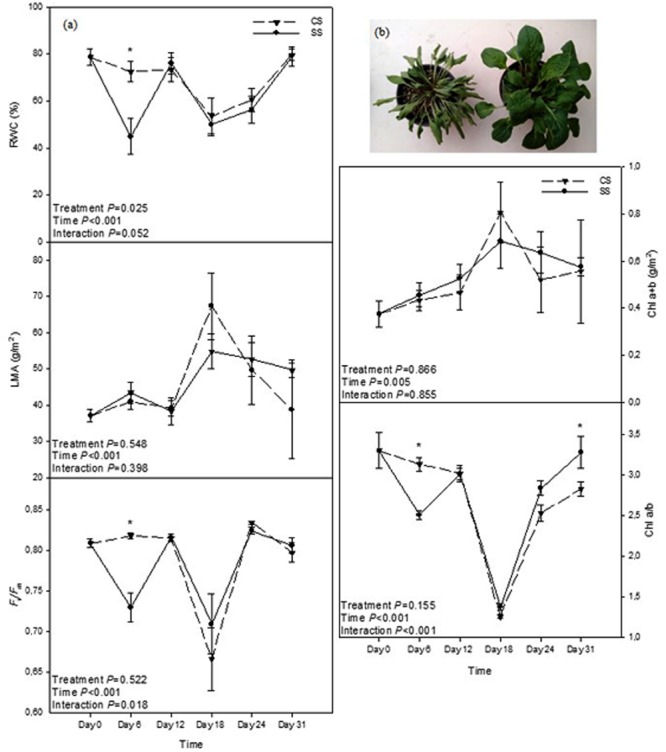
**(a)** Relative water content (RWC), leaf mass per area ratio (LMA), *F*_v_/*F*_m_ ratio, chlorophyll (Chl) a + b and the ratio Chl a/b in leaves of *S. dioica* exposed to reiterated drought (double-stressed – SS – plants) or exposed to water deficit only once (single-stressed – CS – plants). Data represent the mean ± SE of *n* = 6–8 individuals. Significant differences between groups were tested by two-way analysis of variance (ANOVA) and Tukey *post hoc* tests (*P* < 0.05). An asterisk indicates significant differences between SS and CS plants. **(b)** Note that water deficit dramatically alters plant structure, showing the inevitable effects of drought. Aside from changes in the composition of the photosynthetic apparatus, severely stressed plants, as those shown here *(left*), will respond in a different way to controls (*right*) when challenged with a subsequent stress.

Beyond the “omics” approaches, there are, however, other aspects that should be considered in the study of plant responses to reiterated drought. These include structural changes, which in turn are the result of changes in “omics” during previous stress exposure and will severely affect the “omics” and overall physiological response during subsequent stress episodes. By exposing *Silene dioica* plants to reiterated drought in a greenhouse (including two cycles of 6 days of water deficit by withholding water, followed by subsequent periods of six days of recovery), it was found that, despite the relative water content (RWC), LMA, maximum efficiency of the photosystem II (*F*_v_/*F*_m_ ratio) and the total amount of chlorophylls (Chl a + b) not differing between double-stressed and single-stressed plants (SS and CS, respectively), the Chl a/b ratio was higher in SS plants than in CS plants at the end of the experiment (**Figure 1**). It is interesting to note that changes in the Chl a/b ratio were only observed after recovery; this suggests a change in the structure of the photosynthetic apparatus, since it has been reported that there is a reduction in the size of the light harvesting complex of the photosystem II (LHCII) under excess light (Čajánek et al., 1999; Kurasová et al., 2000, 2002). This change in the pigment composition of leaves is therefore indicative of a reduction of the pigment antenna size in double-stressed plants, which might help plants to reduce ROS production and photo-oxidative stress in chloroplasts, if they are challenged by a new stress in the future.

**FIGURE 2:**
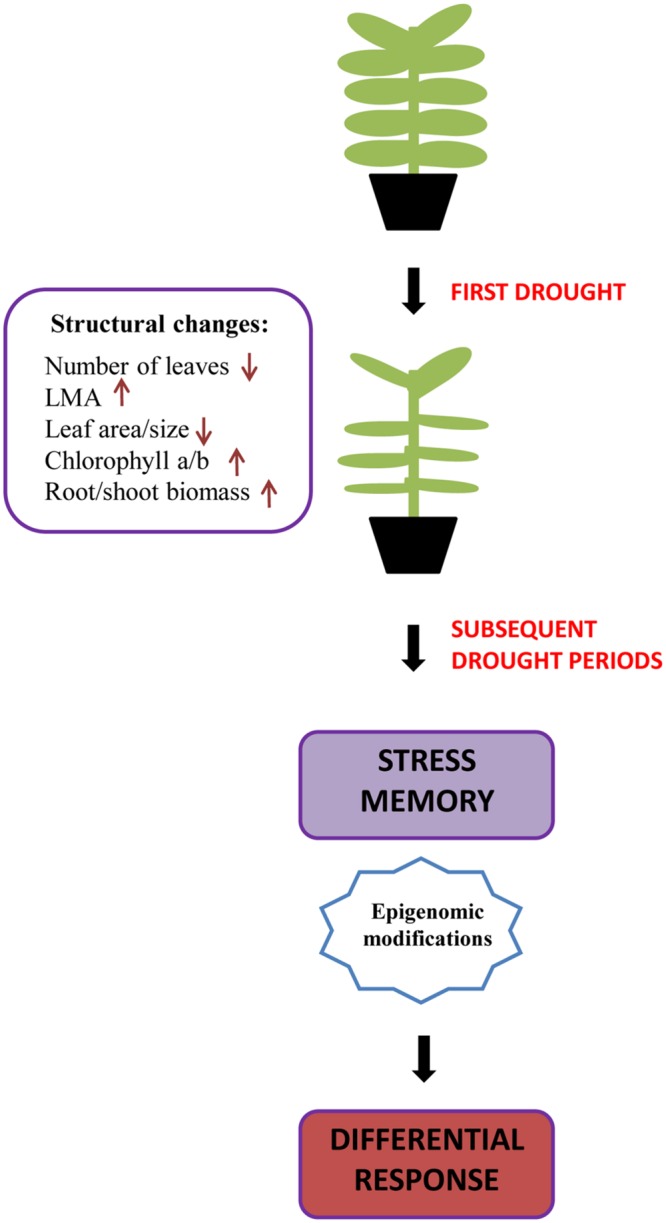
**Diagram to illustrate the biological significance of structural changes in plant responses to reiterated drought.** Stress memory effects that lead to a differential response to repeated drought periods is not only determined by epigenomic changes, but also by the structural changes caused as a result of the first drought.

These results are just an example of structural changes, in this case in the photosynthetic apparatus, as a stress imprint that prepares the plant to respond better to a subsequent period of drought. There are, however, other structural changes that have been reported to occur in plant responses to drought stress in various species. These include a decrease in the leaf area and size, and reductions in the shoot/root ratio in *Quercus ilex* (Peña-Rojas et al., 2004, 2005); a decrease in the number of leaves in *Saccharum* sp. (Zhang et al., 2014); a change in the distribution of roots moving toward the lower layers of the soil in search for water in oaks (Kuster et al., 2012); and even changes in the chloroplast structure and position within the cell in sugarcane (Zhang et al., 2014). Although it has still to be determined to what extent these structural changes contribute to drought stress memory, we propose a model in which structural changes may constitute a stress imprint with significant effects on the plant response to reiterated drought (**Figure 2**). To what extent some changes occur or not will undoubtedly depend not only on the species, but also on the duration and severity of the stress to which the plants are exposed. For instance, Walter et al. (2010) showed that severe drought in grasses not only resulted in biomass loss, but also in reductions in photosynthesis and photoinhibition of the photosynthetic apparatus when plants were challenged by a second drought. Therefore, severe stress in double-stressed plants may result in negative effects; but it is well known that acclimation to small periods of water deficit and/or some water shortage can help improve water use efficiency in ornamental plants and this constitutes a general practice in horticulture (Davies et al., 1992). A first exposure to drought will have inevitable effects on plant structure and function. However, if the stress is not too severe and the plant can recover, it may then respond better to subsequent stresses by showing not only epigenomic changes but also by deploying a different physiological response related to the new adapted structure. This may involve overall reduced transpiration at the whole-plant level, due to reduced size, or changes in photosynthesis and photoprotection, due to an altered pigment composition of the leaves, among a plethora of other possible effects resulting from the first drought. Therefore, the inevitable effects of the first drought can serve to improve the physiological response to reiterated drought.

## Conclusion

Drought is one of the abiotic stresses that most severely affects plant growth and development; consequently, plants rapidly adjust their structure, metabolism and function to withstand it. Nowadays, “omics” approaches, such as epigenomics, transcriptomics, metabolomics, and transcriptomics, provide us with a unique opportunity to solve the complex but at the same time fascinating puzzle of plant responses to drought stress. Combining such approaches with the study of structural changes at various levels of organization (from histone modifications to changes at the whole-plant level) will undoubtedly contribute to our understanding of the mechanisms underlying drought stress memory. An integrated approach is therefore encouraged in future studies of plant responses to reiterated drought to help us understand general water management practices in plant production.

## Author Contributions

Conceived and designed the experiments: EF-S and SM-B. Performed the experiments: EF-S. Analyzed the data: EF-S and SM-B. Contributed reagents/materials/analysis tools: SM-B. Wrote the paper: EF-S and SM-B.

## Conflict of Interest Statement

The authors declare that the research was conducted in the absence of any commercial or financial relationships that could be construed as a potential conflict of interest.
